# Human Health Risk Assessment of Chlorinated Hydrocarbons in Groundwater Based on Multi-Pathway Analysis

**DOI:** 10.3390/toxics12120894

**Published:** 2024-12-09

**Authors:** Yidi Wang, Guilan Li, Xiaohan Li, Ye Yang, Kaifang Ding, Shilu Xing, Yilong Zhang, Luxing Zhang

**Affiliations:** 1 School of Resource and Environmental Engineering, Inner Mongolia University of Technology, Hohhot 010051, China; wangyidi2202@163.com (Y.W.);; 2 Institute of Hydrogeology and Environmental Geology, Chinese Academy of Geological Sciences, Shijiazhuang 050061, China; 3 School of Environmental Science and Engineering, Southern University of Science and Technology, Shenzhen 518055, China

**Keywords:** chlorinated hydrocarbons, multi-pathway, risk assessment, environmental pollution

## Abstract

The rapid development of the global chemical industry has led to widespread groundwater contamination, with frequent pollution incidents posing severe threats to water safety. However, there has been insufficient assessment of the health risks posed by chlorinated hydrocarbon contamination in groundwater around chemical industrial parks. This study evaluates the chlorinated hydrocarbon contamination in groundwater at a chemical park and conducts a multi-pathway health risk assessment, identifying the key risk pollutants. In addition, sensitivity analysis of the primary exposure pathways was performed using the Monte Carlo method. The results indicate severe exceedance of pollutant concentrations with widespread diffusion. Carcinogenic risks were mainly driven by vinyl chloride, whose oral cancer slope factor was significantly higher than that of other substances, while non-carcinogenic risks were dominated by trichloro-ethylene, which had the lowest reference dose. Both carcinogenic and non-carcinogenic risks through the drinking water pathway accounted for approximately 90% of the total risk, whereas the risk contribution from dermal contact was negligible. Although boiling water can partially reduce the risks, its effect on high-concentration pollutants is limited. Additionally, sensitivity analysis showed that pollutant concentration was the primary influencing factor for risk values, followed by exposure duration. The findings of this study provide a scientific basis for effectively formulating pollution control measures and ensuring the drinking water safety of nearby residents.

## 1. Introduction

Industrial site contamination is a critical research area, particularly in China, where both research and management in this field are still in their infancy but are gaining increasing attention [1,2,3]. In 2014, the Ministry of Environmental Protection of China issued the “Technical Guidelines for Risk Assessment of Contaminated Sites” [4], underscoring the need for scientific management of contaminated industrial sites. Chlorinated hydrocarbons, as common industrial compounds, are frequently released into groundwater through improper disposal, resulting in significant environmental and health risks. With groundwater serving as the primary drinking water source for approximately 70% of China’s population [5], chlorinated hydrocarbon contamination has become a serious threat to public health [6].

Globally, groundwater contamination by organic pollutants, particularly chlorinated hydrocarbons, is becoming an increasingly serious issue. Chlorinated hydrocarbons, such as trichloromethane, vinyl chloride, and trichloroethylene, are persistent toxic pollutants that can cause central nervous system damage, liver and kidney damage, and an increased risk of cancer through inhalation, ingestion, or dermal contact. In countries like the United States and Japan, compounds such as trichloroethylene (TCE) and tetrachloroethylene (PCE) often exceed WHO drinking water standards [7,8]. Similarly, in China, cities like Tianjin, Taiyuan, and Zhengzhou have reported severe organic contamination, with many areas having shallow groundwater unsuitable for direct consumption [9,10]. Certain chlorinated hydrocarbons (such as trichloroethylene and vinyl chloride) are classified as Group 1 or 2B carcinogens by the International Agency for Research on Cancer (IARC), highlighting the significant health risks posed by these pollutants.

Chlorinated hydrocarbons in groundwater pose health hazards not only through direct oral ingestion but also through dermal contact or upward migration into the atmosphere, leading to chronic inhalation exposure [11,12]. To minimize remediation costs and protect human health and the environment, researchers have evaluated the carcinogenic and non-carcinogenic risks of these compounds through human health risk assessments (HHRAs). For example, Xiong et al. [13] investigated the environmental levels of volatile organic compounds (VOCs), their potential sources, and associated health risks in two coastal cities in the Vancouver area from 2012 to 2016. Han et al. [14] assessed the health risks of VOCs in groundwater at a contaminated site in Jiangsu Province and reported that trichloromethane inhalation could lead to unacceptable risks. Kawabe et al. [15] reported that the risk levels of perchloroethylene (PCE), trichloroethylene (TCE), and cis-1,2-dichloroethylene (cis-1,2-DCE) in groundwater from contaminated sites in Japan unexpectedly increased at certain times. Additionally, some scholars have analyzed health risks across different exposure pathways, evaluating the carcinogenic and non-carcinogenic risks of various chlorinated hydrocarbons [16].

However, the current health risk assessments for chlorinated hydrocarbons are insufficient [17,18]. Most studies have focused on disinfection byproducts like trihalomethanes, while the risks associated with other chlorinated hydrocarbons remain understudied. Furthermore, traditional deterministic methods for health risk assessments often use fixed input parameters, leading to a potential overestimation or underestimation of the risks. Monte Carlo simulations offer an alternative approach, enabling sensitivity analysis of exposure parameters and providing a more robust basis for risk management [19,20]. Despite these advances, there is limited research on multi-pathway health risk assessments and key pollutant identification for chlorinated hydrocarbons.

This study focuses on a chemical industrial park, systematically evaluating groundwater contamination by 10 chlorinated hydrocarbons and their carcinogenic and non-carcinogenic risks. It also identifies the key risk pollutants and primary exposure pathways, considering local drinking water characteristics. Monte Carlo simulations were used to identify sensitive parameters for high-contribution pathways. The findings provide valuable insights for managing contaminated sites and ensuring drinking water safety.

## 2. Materials and Methods

### 2.1. Overview of the Study Area

**Figure 1:**
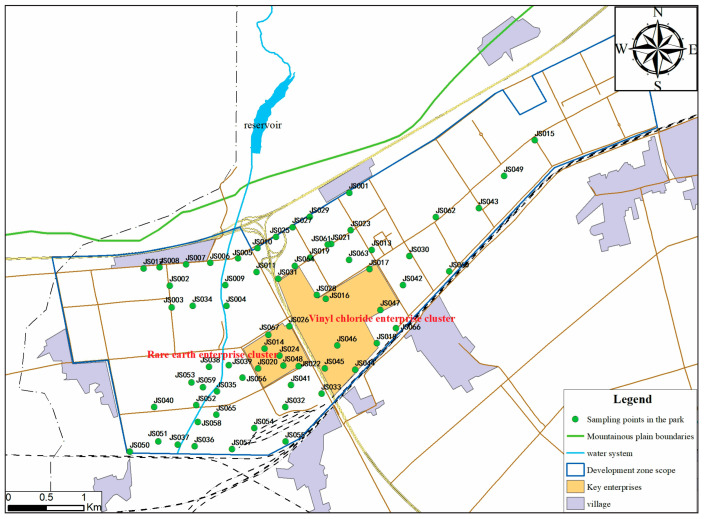
Location of the study area and distribution of monitoring points.

The study area (Figure 1) is located in a continental semi-arid climate zone characterized by dry conditions and significant seasonal climate variations. It lies in the piedmont alluvial fan region, with an overall topography that is higher in the northeast and lower in the southwest. On the basis of the origin of the aquifer system and hydraulic connectivity characteristics, the groundwater in this area is divided into single-structure and double-layer-structure zones. The single-structure zone consists of an unconfined aquifer with hydraulic connections throughout. The double-layer-structure zone is located in the central part of the basin and is characterized by a late Middle Pleistocene silty clay layer. Above the silt layer lies a shallow aquifer, whereas beneath it lies a confined deep aquifer. Water exchange between the shallow and deep aquifers can occur through the silt layer.

The study area hosts multiple chemical enterprises. The western region primarily produces chemical products such as polyvinyl chloride (PVC) and trichloroethylene, with major processes including ion-exchange membrane caustic soda production, PVC production, and desulfurizer production using carbide slag. The eastern part of the study area is occupied mainly by rare earth metal refining enterprises. Additionally, there are many sensitive areas nearby, including centralized drinking water supply sources, scattered rural water supply wells, and residential areas.

### 2.2. Sample Collection and Analysis

The distribution of sampling points for the phreatic monitoring wells is shown in Figure 1 (sampling points numbered JS001–JS0067). A preliminary investigation conducted in 2021 at the site collected 19 groundwater samples, of which 7 were found to contain organic compounds, and 5 samples exceeded the regulatory limits significantly. In this study, groundwater samples were collected between November 2023 and March 2024, with a total of 76 samples analyzed for trichloromethane, vinyl chloride, trichloroethylene, carbon tetrachloride, 1,1-dichloroethane, 1,2-dichloroethane, 1,1,2-trichloroethane, 1,1-dichloroethylene, 1,2-dichloroethylene, and tetrachloroethylene. The sampling was carried out in strict accordance with the Technical Specification for Groundwater Environmental Monitoring to ensure representative coverage of the study area [21].

Water samples were stored in 40 mL amber glass vials with Teflon-lined caps to prevent volatilization losses. Each vial was preserved with 25 mg of ascorbic acid and acidified to a pH ≤ 2 using 0.5 mL of a 1:1 hydrochloric acid solution. The samples were stored at ≤4 °C and transported to the laboratory within 24 h. All analyses were completed within 14 days, following the requirements of the national standard: Determination of Volatile Organic Compounds in Water Using Purge-and-Trap/Gas Chromatography–Mass Spectrometry [22]. Chlorinated hydrocarbons were analyzed using the purge-and-trap/gas chromatography–mass spectrometry (GC–MS) method, in compliance with the technical requirements of HJ 639-2012. The detection limits for the target compounds ranged from 0.2 µg/L to 5.0 µg/L. Strict quality control measures, including procedural blanks and spiked samples, were implemented throughout the process to ensure the accuracy and reliability of the data.

### 2.3. Health and Environmental Risk Assessment Methods for Groundwater

On the basis of China’s HJ25.3-2014 “Technical Guidelines for Risk Assessment of Contaminated Sites” and the U.S. RAGS guidelines, the carcinogenic risk (expressed as RISE) and non-carcinogenic risk (expressed as the hazard quotient, HQ) of chlorinated hydrocarbons in groundwater were calculated. Considering the characteristics of chlorinated hydrocarbons and local groundwater usage by residents, four different exposure pathways have been evaluated: oral ingestion, dermal absorption, indoor inhalation, and outdoor inhalation [23,24,25]. The researchers’ results indicated differences in identifying the main exposure risk pathways, and challenges were also faced in determining the pollutants with the highest risk. Boiling drinking water has been widely recognized as an important “detoxification” process that can reduce health risks caused by volatile halocarbons in water. Considering the local practice of consuming boiled water, the assessment incorporated a conservative estimate of a 60% reduction in chlorinated hydrocarbons after boiling [26,27].

In this study, Monte Carlo simulation was used to conduct a sensitivity analysis of the data. Sensitivity analysis was performed using Oracle Crystal Ball^®^ (version 11.1.34,190, with 10,000 iterations) in Excel software. The parameters used in the Monte Carlo simulation were obtained from extensive local research data [26,28].

## 3. Results and Discussion

### 3.1. Chlorinated Hydrocarbons in Groundwater

In the groundwater samples from the study area, certain concentrations of chlorinated hydrocarbons were detected (Table 1). The detection rates of chlorinated hydrocarbons in groundwater, from highest to lowest, were trichloroethylene, 1,1-dichloroethane, 1,2-dichloroethylene, perchloroethylene, vinyl chloride, 1,1,2-trichloroethane, 1,1-dichloroethylene, trichloromethane, 1,2-dichloroethane, and carbon tetrachloride. Among them, the detection rate of carbon tetrachloride was 11.9%, while that of the other compounds exceeded 40%. According to the national drinking water standards [29], trichloroethylene had the highest exceedance rate at 71.6%, followed by vinyl chloride (61.2%), 1,1-dichloroethane (55.2%), 1,1,2-trichloroethane (53.7%), 1,2-dichloroethylene (52.2%), 1,1-dichloroethylene (34.3%), perchloroethylene (31.3%), trichloromethane (26.9%), carbon tetrachloride (10.4%), and 1,2-dichloroethane (4.5%). The detailed concentration data and further statistical analysis are provided in Supplementary Table S1.

**Figure 2:**
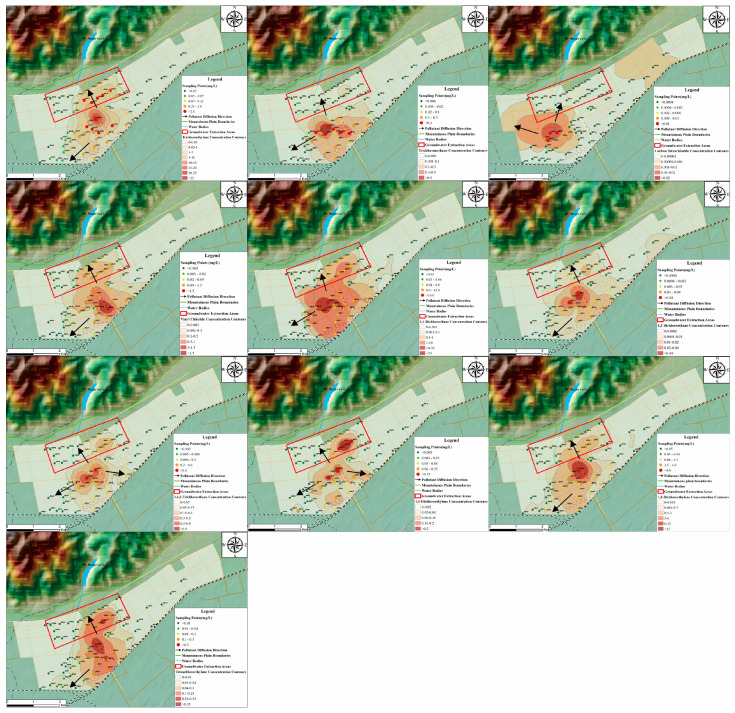
Spatial distribution map of chlorinated hydrocarbon pollutants.

The spatial distribution of chlorinated hydrocarbons (Figure 2) reveals varying contamination patterns for different compounds. Vinyl chloride shows widespread contamination, primarily concentrated in the southwestern part of the study area, with an outward diffusion pattern from the center. This is likely due to regional topography and hydrodynamic conditions that facilitate its rapid migration through groundwater. Trichloroethylene contamination is prominent in the central region, characterized by high concentrations and a wide range, indicating a significant impact from industrial activities. Carbon tetrachloride contamination is relatively localized, primarily in the southeastern corner, with a small diffusion range but high localized concentrations. Other chlorinated hydrocarbons, such as trichloromethane, 1,2-dichloroethane, 1,1-dichloroethylene, tetrachloroethylene, and 1,1,2-trichloroethane, exhibit scattered high-concentration hotspots. Trichloromethane and 1,2-dichloroethane have broader contamination ranges, while 1,1-dichloroethylene and tetrachloroethylene show smaller ranges but higher concentrations, suggesting localized industrial sources. The distribution of 1,1,2-trichloroethane is more dispersed, concentrated in specific areas.

Most contamination hotspots are associated with industrial activities, particularly in the southwestern and central parts of the study area. To mitigate the long-term ecological impacts of pollution, priority should be given to addressing contamination hotspots in urbanized and industrialized areas [30]. Vinyl chloride and trichloroethylene represent the most critical pollutants, with widespread and high-concentration hotspots posing significant risks to groundwater quality. Contaminant diffusion patterns are circular or elliptical, likely influenced by pollution sources and groundwater flow directions. Studies have shown that in porous media with high hydraulic conductivity, contaminants tend to diffuse in the direction of groundwater flow while migrating downward [31]. Although contamination is currently localized, continued migration downstream, combined with the influence of extraction processes, could affect broader areas, underscoring the urgent need for remediation measures to mitigate health risks and protect groundwater resources.

### 3.2. Multi-Pathway Assessment of Chlorinated Hydrocarbons

Due to the lack of key parameters, the respiratory unit cancer factors for 1,1-dichloroethylene and 1,2-dichloroethylene are missing. Since their carcinogenic potential for humans has not been fully evaluated, it is not possible to assess their carcinogenic risks comprehensively. Generally, in carcinogenic risk assessments, a risk value below 1.00 × 10^−6^ can be considered negligible, whereas a value above 1.00 × 10^−6^ is deemed unacceptable. In non-carcinogenic risk assessments, an HQ value less than 1 indicates that adverse health effects are unlikely, whereas an HQ value greater than 1 suggests a potential risk to health [32].

#### 3.2.1. Carcinogenic and Non-Carcinogenic Risk Values

**Figure 3:**
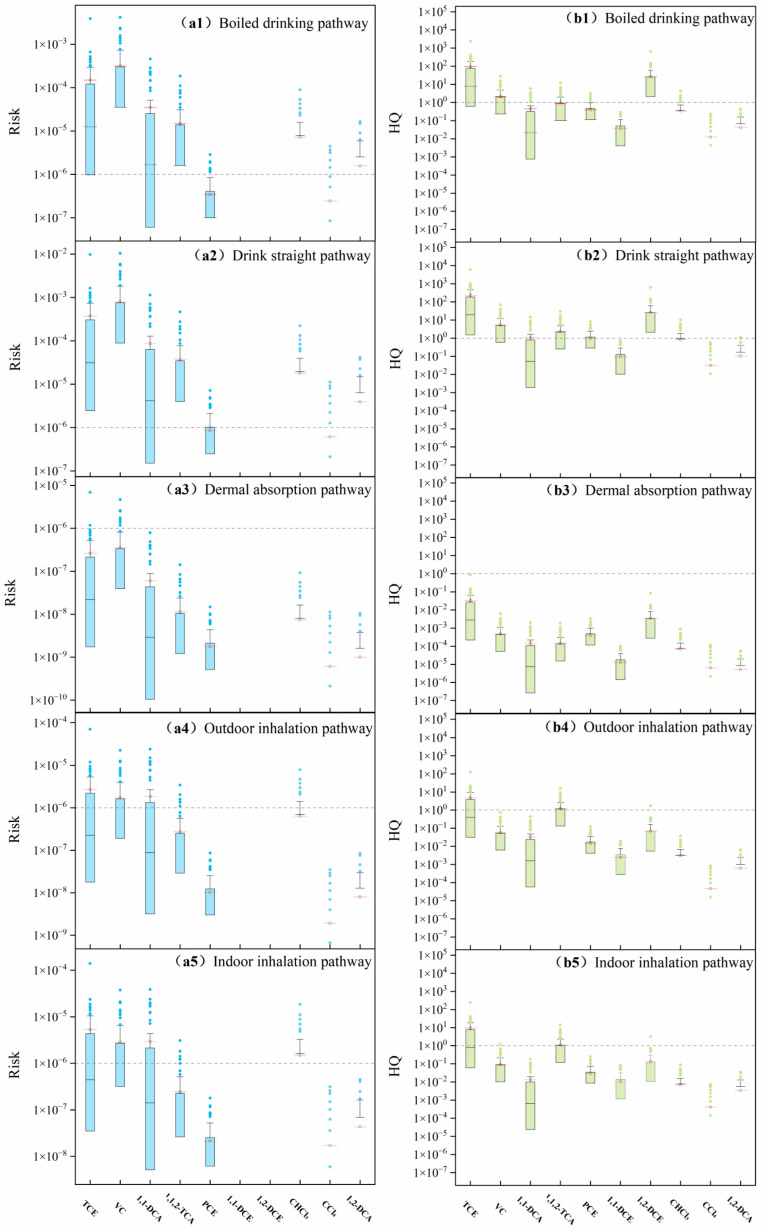
Risk values and hazard quotients of chlorinated hydrocarbons through different exposure pathways. (Risk values for chlorinated hydrocarbons in different exposure routes in blue; hazard quotients for chlorinated hydrocarbons in different exposure routes in green).

Overall, the risk values of various pollutants vary significantly across different exposure pathways (Figure 3). Among the four exposure pathways, drinking water poses the highest carcinogenic risk to human health. In the drinking water pathway, vinyl chloride has the highest carcinogenic risk, with all detected risk values exceeding 1.00 × 10^−6^, and the highest risk, 0.01, poses a serious health threat. The median risk values for vinyl chloride, trichloroethylene, and 1,1,2-trichloroethane exceed the permissible limits, whereas some data for 1,1-dichloroethane, perchloroethylene, trichloromethane, carbon tetrachloride, and 1,2-dichloroethane also exceed the limits. Most of the median risk values for these substances are approximately 1.00 × 10^−5^, indicating a significant carcinogenic risk from drinking directly. The average ranking of carcinogenic risk in drinking water is as follows: vinyl chloride > trichloroethylene > 1,1-dichloroethane > 1,1,2-trichloroethane > trichloromethane > 1,2-dichloroethane > 1.00 × 10^−6^ > perchloroethylene > carbon tetrachloride.

Studies have shown that the primary risk from chlorinated hydrocarbon exposure comes from oral ingestion, followed by inhalation and dermal absorption (oral > inhalation > dermal absorption) [33,34]. The percentage contribution analysis indicated that oral ingestion accounted for more than 97% of the total cancer risk from THM exposure, followed by inhalation (2.32%) and dermal absorption (0.02%). The high risk from oral ingestion is likely linked to water consumption, as drinking water is one of the most common activities in human life. However, some studies suggest that inhalation is the primary pathway for human risk exposure [35]. The difference in the ranking of exposure pathways may be due to variations in pollutant concentrations and distributions in groundwater. Further research is needed to resolve this discrepancy and identify the primary risk pathways for chlorinated hydrocarbon exposure.

Although the literature indicates that boiling water can reduce pollutant concentrations by approximately 60%, its impact on the carcinogenic risk of high-concentration pollutants remains limited. In Figure 3(a1), the average risk values for trichloroethylene, vinyl chloride, trichloromethane, and 1,1-dichloroethane after boiling still exceed the national regulatory limits.

The carcinogenic risk values for dermal contact are significantly lower than those for other pathways, with almost all the data falling below the regulatory limits and only a few risk values for trichloroethylene and vinyl chloride exceeding 1.00 × 10^−6^. This suggests that among the four pathways, dermal contact poses the smallest carcinogenic risk, likely because of the shorter contact time and lower carcinogenic parameters associated with this exposure route. The median values for dermal contact are mostly lower than the average, indicating that chlorinated hydrocarbon concentrations are relatively low in many areas, with higher concentrations concentrated in specific regions. The average risk ranking is as follows: 1.00 × 10^−6^ > vinyl chloride > trichloroethylene > 1,1-dichloroethane > 1,1,2-trichloroethane > trichloromethane > perchloroethylene > 1,2-dichloroethane > carbon tetrachloride.

The carcinogenic risk from inhalation can be divided into indoor and outdoor exposures, with both showing similar risk trends. However, the carcinogenic risk values for indoor inhalation are slightly higher than those for outdoor inhalation, indicating that inhaling polluted air in enclosed environments may lead to significant carcinogenic risks. As shown in the figure, the average risk values for trichloroethylene, vinyl chloride, and 1,1-dichloroethane exceed the regulatory limits. This finding aligns with the research by Zhu et al. [11], which also confirmed that indoor air pollution can result in higher health risks, particularly when inhaling volatile organic compounds (VOCs). Specifically, the carcinogenic risks of substances such as trichloroethylene, vinyl chloride, and 1,1-dichloroethane often exceed regulatory limits in indoor inhalation pathways, which is consistent with multiple studies. Similarly, the carcinogenic risk values for outdoor inhalation followed the same pattern, with the average risk for trichloromethane also exceeding the limit, suggesting that trichloromethane may also pose a carcinogenic risk in outdoor environments.

The hazard quotient (HQ) follows a similar pattern to the risk values, showing significant variation across different pathways for chlorinated hydrocarbons. The distribution of HQs for different substances within the same pathway is broad, primarily due to variations in pollutant concentrations. Although boiling water can reduce pollutant concentrations to some extent, similar to the risk values, its impact on the final HQ is limited. The maximum HQ values for trichloroethylene and 1,1-dichloroethylene can exceed 1000, and for direct consumption, most HQs are higher than the acceptable limits. In the drinking water pathway, trichloroethylene, vinyl chloride, and 1,1-dichloroethylene pose substantial health risks. This finding is consistent with Singha et al. [36], who also found high HQ values in the drinking water pathway, particularly in areas with high concentrations of specific pollutants. However, in contrast to this study, Singha et al. observed higher HQ values for inhalation exposure as well.

Dermal contact remains the pathway with the lowest hazard, as the HQ values are generally low and do not exceed the limits, indicating that even in extreme cases, the non-carcinogenic risks associated with dermal contact are minimal. With respect to inhalation, trichloroethylene and 1,1,2-trichloroethane are the greatest hazards. Additionally, indoor inhalation presents a greater hazard than outdoor inhalation does.

#### 3.2.2. Contribution Rate and Standardization Analysis

When the risk values across different pathways are calculated, the proportion of each pathway for the same substance remains constant, which may be determined by the substance itself [16]. An analysis of the formulas across pathways clearly reveals that the proportions eliminate the influence of the pollutant concentration during calculation but are closely related to various intrinsic factors of the substance (e.g., the carcinogenic slope factor and diffusion coefficient). However, the current studies on chlorinated hydrocarbons in human health risk assessments reveal differing conclusions: some studies suggest that inhalation is the primary exposure pathway, whereas others argue that ingestion is the main route. This discrepancy may be due to greater factors for certain pollutants in these pathways, the intrinsic characteristics of the substances, and the parameter values selected by researchers.

**Figure 4:**
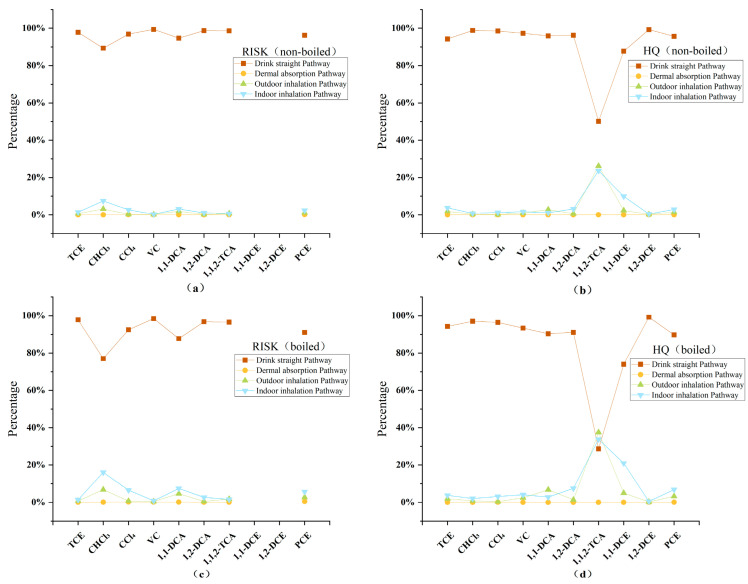
Contribution rates of various chlorinated hydrocarbons across different pathways (including risk values and hazard quotients). (**a**,**b**) are the risk values and hazard quotients before boiling, while (**c**,**d**) are the risk values and hazard quotients after boiling.

In terms of the carcinogenic risk values, the ingestion pathway accounts for the vast majority, with vinyl chloride contributing up to 99.39% and trichloromethane the lowest at 89.34% (Figure 4). Some researchers have reported that the carcinogenic risk from the oral ingestion of chlorinated hydrocarbons in chemical plants accounts for 98.41% to 99.69% of the total carcinogenic risk, whereas inhalation and dermal contact account for 0.03% to 1.33% and 0.27% to 0.38%, respectively [37]. This is related to the fact that exposure through the ingestion pathway is at least 10 times greater than that through other pathways. Chlorinated hydrocarbons are more efficiently absorbed when ingested through drinking water, while inhalation is influenced by the volatilization factor of the pollutants in the air, and dermal contact is associated with the skin permeability coefficient, resulting in much lower exposure than ingestion. Other studies have also shown that the carcinogenicity of chlorinated hydrocarbon pollutants through the drinking water pathway is significantly greater than that through other exposure pathways [16,28,38]. The impact of dermal contact remains minimal, with risk values remaining below 0.2%. The hazard quotient (HQ) trend is generally similar, but for 1,1,2-trichloroethane, the HQ for the ingestion pathway significantly decreases, whereas the proportion for inhalation increases. As mentioned previously, although boiling water can reduce some of the risk values, the carcinogenic risk for high-concentration pollutants such as trichloroethylene remains high, and the risk associated with the ingestion pathway is far from eliminated [39].

**Figure 5:**
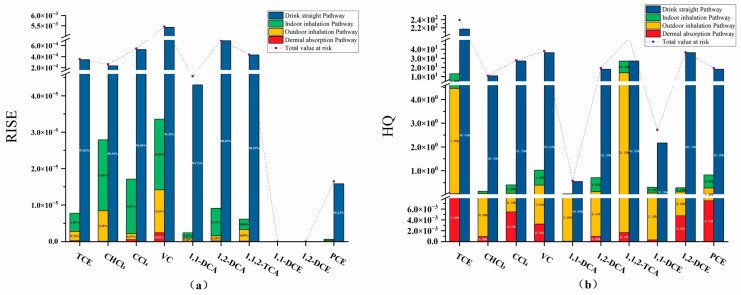
Standardized human health risk values (**a**): carcinogenic risk values, (**b**): non-carcinogenic hazard quotients).

To ensure comparability across pollutants with varying concentrations, all substance concentrations were standardized to 1 mg/L before calculating the associated risk values. Figure 5 illustrates the contributions of different exposure pathways to both the total carcinogenic risk (RISE, Figure 5a) and the hazard quotient (HQ, Figure 5b). The results show that ingestion is the dominant exposure pathway for all pollutants, accounting for over 90% of the total risk in most cases. Among them, vinyl chloride exhibits the highest ingestion-related risk at 99.39%, while trichloromethane has the lowest at 89.34%. These findings align with numerous studies, which indicate that ingestion of contaminated drinking water is the greatest health threat, further confirming that ingestion is the primary contributor to carcinogenic risk [16,37,39].

In contrast, the contribution of inhalation to the overall risk is smaller, although it remains more significant for volatile pollutants such as trichloroethylene and vinyl chloride. This can be attributed to the higher volatility of these pollutants, which enables them to evaporate from water into the air, thus increasing inhalation exposure. Dermal contact, on the other hand, contributes the least to the total risk across all pollutants, with values consistently below 0.2%. This is consistent with the existing literature, which suggests that skin exposure has a minimal impact on the health risks associated with most pollutants [40]. An interesting deviation is observed for 1,1,2-trichloroethane, where the HQ for ingestion decreases, while inhalation accounts for a relatively higher proportion compared to other pollutants. Furthermore, trichloroethylene remains a high-risk pollutant through ingestion, even when mitigation measures, such as boiling water, are applied. This suggests that conventional water treatment methods may not effectively remove certain pollutants.

The standardized trends observed in the risk values and HQs are in good agreement with field test data, reinforcing the dominance of ingestion as the primary exposure pathway. When compared to similar studies in the literature, such as those by Liu et al. [16] and Kujlu et al. [41], which also demonstrate that ingestion is the most significant pathway for the health risks associated with most pollutants, the results of this study are further validated. These findings underscore the urgent need for targeted remediation strategies focused on reducing ingestion-related exposure, particularly for pollutants like vinyl chloride and trichloroethylene. Effective water treatment methods are essential to mitigate the health risks posed by these contaminants.

### 3.3. Analysis of Key Pollutants in Risk Assessment

**Figure 6:**
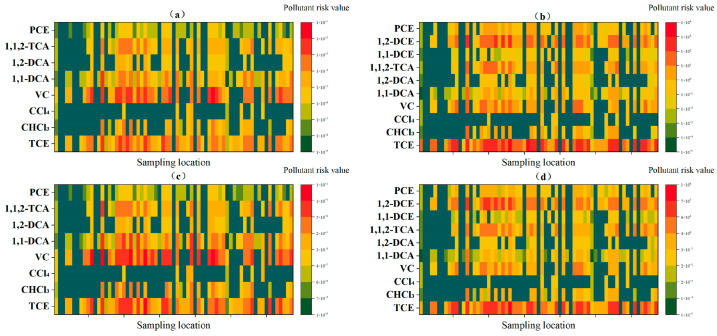
Pollutant risk values at different sampling locations. (**a**) Carcinogenic risk values for direct drinking; (**b**) Non-carcinogenic hazard quotients for direct drinking; (**c**) Carcinogenic risk values for boiled water consumption; (**d**) Non-carcinogenic hazard quotients for boiled water consumption.

The current research on chlorinated hydrocarbons has focused primarily on disinfection byproducts, whereas studies on chlorinated hydrocarbons in chemical plants remain limited, and related risk analyses and key pollutant discussions are insufficient [39,41]. Figure 6 shows the risk values of chlorinated hydrocarbon pollutants at each sampling point. The carcinogenic risk values indicate that vinyl chloride has higher values at most locations, followed by trichloroethylene, whereas carbon tetrachloride has a lower detection rate. For non-carcinogenic hazard quotients, trichloroethylene generally has higher values, followed by 1,2-dichloroethylene. The proportions of pollutants with carcinogenic and non-carcinogenic risks vary, which is related to the nature of the pollutants. Carcinogenic risk is primarily determined by the carcinogenic slope factor (SF), which indicates an increased probability of cancer per unit dose exposure. Different pollutants have varying carcinogenic slope factors, with vinyl chloride and trichloroethylene having the highest oral carcinogenic slope factors. Compared with the other pathways, the ingestion pathway has a more significant impact. The non-carcinogenic risk, on the other hand, is based on the reference dose (RfD), which is the dose at which no significant health effects are expected over a lifetime of exposure. Different pollutants have varying reference doses, and these reference doses also differ for different body systems. Since the hazard quotient is inversely proportional to the reference dose, a smaller reference dose results in a greater hazard. The oral reference dose for trichloroethylene is the lowest, whereas vinyl chloride and 1,2-dichloroethylene have the same reference dose. However, due to the lower concentration of vinyl chloride pollutants, their contribution to the overall risk is minimal.

On average (Supplementary Table S2), the types of chlorinated hydrocarbons contributing the most to the carcinogenic risk values and non-carcinogenic hazard quotients remain consistent, regardless of whether the water is boiled or not boiled, with similar proportions. In the case of direct drinking, vinyl chloride dominated the carcinogenic risk, contributing 59.8%, whereas trichloroethylene accounted for 86.2% of the non-carcinogenic hazard quotient. When boiled water is consumed, vinyl chloride remains the main contributor to carcinogenic risk, reaching 58.9%, whereas trichloroethylene continues to dominate the non-carcinogenic hazard quotient, contributing 76.3%.

In conclusion, vinyl chloride plays a dominant role in determining the carcinogenic risk in the groundwater at this chemical site, whereas trichloroethylene has a key influence on the non-carcinogenic hazard quotient. To eliminate the interference of concentration differences, all pollutant concentrations were standardized to 1 mg/L. After standardization, the results were consistent with those before standardization; however, the contribution proportions changed: the contribution of vinyl chloride to carcinogenic risk increased to 69.9% and to 69.1% when boiling was considered. For the non-carcinogenic hazard quotient, trichloroethylene contributed 52.7%, and 49.7% of the non-carcinogenic hazard quotient after boiling was considered.

### 3.4. Sensitivity Analysis

Due to the high weight of the drinking water pathway in the risk assessment model, a sensitivity analysis was conducted in this study to evaluate each parameter’s contribution under the drinking pathway, which is crucial for identifying the key influencing factors. A sensitivity analysis based on the Monte Carlo model identified the primary factors affecting risk. The analysis results indicate that a positive sensitivity value indicates a positive correlation with risk, whereas a negative value indicates a negative correlation. The larger the sensitivity value is, the more significant the factor’s impact on the risk value [42].

**Figure 7:**
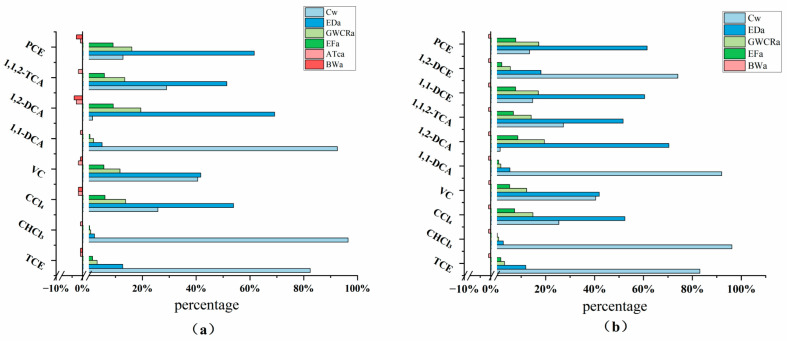
Sensitivity analysis of drinking water pathway based on Monte Carlo simulation ((**a**) represents the sensitivity analysis for carcinogenicity, and (**b**) represents the sensitivity analysis for non-carcinogenicity).

Overall, the sensitivities of the carcinogenic risk values and non-carcinogenic hazard quotients are quite similar (Figure 7). For most pollutants, the concentration of contaminants in drinking water is the key variable affecting health risk. The concentrations of trichloroethylene, trichloromethane, 1,1-dichloroethane, and 1,2-dichloroethylene account for the highest proportion of both the carcinogenic risk values and hazard quotients, ranging from 74.1% to 96.5%. Compared with other pathways, drinking water, which is the most direct exposure route, results in higher exposure levels and greater health impacts. Studies by Liu et al. [16] and Swartjes [39] also confirm the dominant role of the drinking water pathway in both carcinogenic and non-carcinogenic risks, emphasizing the decisive influence of pollutant concentrations in drinking water on health risks. Furthermore, Kujlu et al. [41] highlighted that high concentrations of trichloroethylene and vinyl chloride in drinking water significantly increase both the non-carcinogenic and carcinogenic risks, which is consistent with the findings of this study. Zhu et al. (2024) further discussed the long-term health impacts of chlorinated hydrocarbon exposure in aquatic environments, stressing the need for improvements in water quality and pollution control measures. In addition to pollutant concentration, exposure duration (ED) is also a crucial variable, accounting for 40% to 70% of the remaining six chlorinated hydrocarbons. These findings indicate that long-term exposure to these chemicals is a key factor in increasing their health risks [43]. Han et al. [14] noted that dermal contact contributes minimally to the exposure risk for most pollutants, especially when these pollutants are primarily exposed via air or water. The shorter exposure durations of the dermal contact and inhalation pathways limit their cumulative risk, making the contribution of the drinking pathway more significant. Daily drinking water consumption (GWCR) and exposure frequency (EF) are secondary variables, and for all pollutants, the influence of exposure frequency is smaller than that of water consumption. The average time for carcinogenic effects and average body weight have a minor impact and are negatively correlated.

The concentration of pollutants in drinking water and exposure duration are key variables affecting the health risks posed by different pollutants. However, for certain chemicals (e.g., 1,2-dichloroethane and perchloroethylene), daily drinking water consumption (GWCR) also plays a significant role. Overall, exposure duration (ED) and pollutant concentration (Cw) have the greatest impacts on overall health risk, with contribution rates ranging from 70% to 99%. These findings are consistent with the research of several scholars. Zhu et al. [11] pointed out that the drinking water pathway dominates the exposure to most pollutants, and exposure duration significantly contributes to the non-carcinogenic hazard quotient (HQ). Maurice et al. [34] emphasized the impact of long-term exposure on health risks from chemical pollutants, a conclusion that is also validated by the results of this study.

By calculating the risk control values for the key influencing parameters, the pollutant concentration risk control values and exposure duration risk control values at average concentrations were obtained (Supplementary Table S3). For the pollutant concentration risk control values, the lowest carcinogenic risk was for vinyl chloride at 0.00018 mg/L, whereas for non-carcinogenic risk, trichloroethylene had the lowest concentration at 0.00461 mg/L. At average concentrations, an exposure duration for vinyl chloride exceeding 0.032a would surpass the carcinogenic risk threshold, whereas for trichloroethylene, an exposure duration exceeding 0.108a would exceed the non-carcinogenic risk threshold.

## 4. Conclusions

This study investigated the contamination of groundwater surrounding a chlorinated hydrocarbon chemical plant, and the exceedance rates were as follows: trichloroethylene (71.6%), vinyl chloride (61.2%), 1,1-dichloroethane (55.2%), 1,1,2-trichloroethane (53.7%), 1,2-dichloroethylene (52.2%), 1,1-dichloroethylene (34.3%), tetrachloroethylene (31.3%), trichloromethane (26.9%), carbon tetrachloride (10.4%), and 1,2-dichloroethane (4.5%). Among these, trichloroethylene exhibited the highest exceedance, with a maximum concentration of 28 mg/L. Spatial distribution analysis indicated that chlorinated hydrocarbon pollutants were primarily concentrated in the southwestern and central areas of the study region, forming significant pollution hotspots with a potential risk of downstream migration.

A multi-pathway exposure analysis and key risk pollutant analysis were conducted on the basis of the human health risk assessment of 10 chlorinated hydrocarbons. The results indicate that the drinking water pathway is the primary exposure route for health risks, with contribution rates mostly exceeding 90%. For carcinogenic risk, vinyl chloride contributes the most; for non-carcinogenic hazard quotients, trichloroethylene is the main contributor, mainly because of the different influencing factors between the two.

For most pollutants, the concentration of pollutants in drinking water is the most critical factor influencing health risks, followed by exposure duration, with a combined contribution rate of 70% to 99%. For pollutant concentrations, when the vinyl chloride concentration exceeds 0.00018 mg/L, the carcinogenic risk surpasses the control value; under non-carcinogenic conditions, trichloroethylene should not exceed 0.00461 mg/L. At average concentrations, the exposure duration for vinyl chloride should not exceed 0.032a for carcinogenic risk, while for non-carcinogenic risk, trichloroethylene exposure should not exceed 0.108a. On the basis of the results of this study, the following risk mitigation measures can be recommended: (a) completely avoid oral exposure and (b) minimize exposure time if water use cannot be prevented. In addition, environmental regulators and risk assessors should focus on regularly collecting health data from residents in the affected area to establish a link between actual concentrations and suspected toxic health effects. This will help expedite the remediation of areas affected by groundwater and guide the development of policies and frameworks to implement regulatory standards.

## Figures and Tables

**Table 1:** Basic information on the pollutants.

	TCE	CHCl₃	CCl₄	VC	1,1-DCA	1,2-DCA	1,1,2-TCA	1,1-DCE	1,2-DCE	PCE
Overstandard rate	71.6%	26.9%	10.4%	61.2%	55.2%	4.5%	53.7%	34.3%	52.2%	31.3%
Detection rate	82.1%	41.8%	11.9%	62.7%	80.6%	40.3%	61.2%	56.7%	71.6%	65.7%
Maximum (mg/L)	28	0.95	0.021	1.92	26.5	0.06	1.08	0.33	17.6	0.45
Average value (mg/L)	1.07	0.08	0.00	0.15	2.01	0.01	0.09	0.04	0.72	0.05

## Data Availability

All data generated or analyzed during this study are included in this published article [and its Supplementary Information Files].

## Supplementary Materials

The following are available online at https://www.mdpi.com/article/10.3390/toxics12120894/s1, Table S1: Concentrations of Chlorinated Hydrocarbons in Groundwater (mg/L), Table S2: Mean and Normalized Risk Values of Various Chlorinated Hydrocarbons, Table S3: Risk Control Values for the Parameters.
