# Supplementary material for: Human Health Risk Assessment of Chlorinated Hydrocarbons in Groundwater Based on Multi-Pathway Analysis
Source: Toxics. 2024 Dec 9;12(12):894. doi: 10.3390/toxics12120894 (PMC11728594; doi:10.3390/toxics12120894)
Supplement: Supplementary file 1 [file toxics-12-00894-s001.zip › toxics-3316706-supplementary.pdf]

Their maximum concentrations (average concentrations) are as follows: carbon tetrachloride 0.021 mg/L (0.0012 mg/L), chloroform 0.95 mg/L (0.077 mg/L), 1,1-dichloroethane 26.5 mg/L (2.01 mg/L), 1,2-dichloroethane 0.06 mg/L (0.0057 mg/L), 1,1,2-trichloroethane 1.08 mg/L (0.086 mg/L), vinyl chloride 1.92 mg/L (0.15 mg/L), 1,1-dichloroethylene 0.33 mg/L (0.043 mg/L), 1,2-dichloroethylene 17.6 mg/L (0.72 mg/L), trichloroethylene 28 mg/L (1.07 mg/L), and tetrachloroethylene 0.45 mg/L (0.054 mg/L).

Supplementary Table S1 Concentrations of Chlorinated Hydrocarbons in Groundwater (mg/L)

Name	TCE	$CHCl_3$	CCl_4	VC	1,1-DCA	1,2-DCA	1,1,2-TCA	1,1-DCE	1,2-DCE	PCE
JS001	0.0044	0	0	0	0	0	0	0	0	0
JS002	0	0	0	0	0	0	0	0	0	0
JS003	0	0	0	0	0	0	0	0	0	0
JS004	0.05	0	0	0.0035	0.0179	0	0	0	0.0217	0
JS005	0.0827	0	0	0.0071	0.0096	0	0	0	0.0169	0.003
JS006	0	0	0	0	0	0	0	0	0	0
JS007	0	0	0	0	0	0	0	0	0	0
JS008	0.0075	0	0	0	0.0026	0	0	0	0	0
JS009	0.127	0	0	0.0163	0.0173	0	0	0	0.0545	0.0027
JS010	0.835	0	0	0.058	0.0745	0	0.0146	0.0095	0.1214	0.0384
JS011	2.78	0	0	0.64	0.288	0.0047	0.0121	0.0294	0.852	0.062
JS012	0	0	0	0	0	0	0	0	0	0
JS013	0.0031	0	0	0	0.0066	0	0	0	0	0
JS014	3.75	0.95	0	0.724	26.5	0.054	1.08	0.296	4	0.132
JS015	0.0027	0	0	0	0.0033	0	0	0	0	0
JS016	0.089	0.0027	0	0.0131	0.0286	0	0.0103	0.0047	0.0134	0.0236
JS017	0.0243	0	0	0.0034	0.0183	0	0.0021	0	0.0086	0
JS018	0.394	0.282	0	0.334	1.24	0.02	0.152	0.106	0.326	0.044
JS019	3.1	0.0043	0	0.35	2.45	0.0232	0.35	0.33	1.65	0.31
JS020	1.86	0.56	0.0042	0.165	16.4	0.0166	0.425	0	2.53	0.075
JS021	3.32	0	0	0.195	1.1	0.0106	0.245	0.22	1.41	0.21
JS022	2.1	0.57	0	1.08	13.6	0.0215	0.435	0.0034	2.8	0.18
JS023	0.0753	0	0	0.0077	0.0147	0	0.0026	0.0026	0.221	0.0022
JS024	2.33	0.36	0	0.485	12.9	0.0327	0.5	0.13	3.47	0.12
JS025	0.14	0	0	0.054	0.186	0	0.0166	0.017	0.0608	0.0156
JS026	28	0.11	0	1.03	8.35	0.06	0.64	0.27	17.6	0.45
JS027	0.408	0	0	0.0367	0.446	0.0033	0.05	0.068	0.181	0.05
JS028	0.0778	0	0	0.138	0.0622	0	0.0029	0.0051	0.0574	0.0099
JS029	0.0941	0	0	0.0112	0.097	0	0.0092	0.0059	0.0286	0.0068
JS030	0.0114	0	0	0	0.0044	0	0	0	0	0
JS031	4.74	0	0	0.129	0.968	0.0022	0.0247	0.078	0.634	0.218
JS032	1.54	0.274	0	0.118	4.92	0.0105	0.124	0.102	1.33	0.114
JS033	0.44	0.083	0	0.204	0.666	0.0106	0.08	0.031	0.232	0.118
JS034	0	0	0	0	0	0	0	0	0	0
JS035	0.545	0.125	0.0068	0.0391	5.75	0.009	0.0775	0.0675	0.715	0.023
JS036	0.0566	0.0026	0	0	0.0039	0	0	0	0.0803	0.0338
JS037	0.0395	0	0	0	0	0	0	0.0768	0	0.0235
JS038	0.0244	0.0098	0.0174	0	0.306	0	0.0059	0	0.0303	0

JS039	1	0.354	0.021	0.098	11.5	0.0147	0.202	0.11	1.37	0.0392
JS040	0	0	0	0	0	0	0	0	0	0
JS041	0.87	0.086	0	0.142	1.77	0.0112	0.056	0.057	0.634	0.113
JS042	0.007	0	0	0	0.0291	0	0	0	0	0
JS043	0	0	0	0	0	0	0	0	0	0
JS044	0.199	0.0219	0	0.288	0.364	0.0116	0.044	0.0212	0.103	0.064
JS045	0.718	0.452	0	1.92	1.33	0.0112	0.074	0.058	0.648	0.294
JS046	2.2	0.168	0	0.322	1.7	0.0092	0.0368	0.07	0.814	0.118
JS047	0.168	0.0071	0	0.0262	0.184	0.0042	0.0305	0.0149	0.0677	0.0389
JS048	0.734	0.07	0	0.0174	1.5	0.0046	0.082	0.04	0.552	0.088
JS049	0.0003	0.0008	0.0004	0.0003	0.0002	0.0004	0.0006	0.0003	0.0009	0.0003
JS050	0.0067	0	0	0	0.0035	0	0	0	0	0
JS051	0.0293	0	0	0	0	0	0	0	0.0165	0.0087
JS052	0.0314	0	0	0	0.0392	0	0	0	0.0129	0.0069
JS053	0.0224	0.0061	0.01	0	0.123	0	0.0031	0	0.0046	0.0018
JS054	0.79	0.136	0	0.0251	2.96	0.0069	0.087	0.054	0.769	0.0358
JS055	0.78	0.128	0	0.0425	1.93	0	0.059	0.04	0.95	0.047
JS056	1.45	0.248	0.0024	0.092	8.53	0.0116	0.242	0.079	1.33	0.0353
JS057	0	0	0	0	0.0106	0	0	0	0	0
JS058	0.0581	0	0	0	0.0189	0	0	0	0.0383	0.0156
JS059	0.0288	0	0.015	0.0095	0.306	0	0.009	0.0024	0.0457	0
JS060	0	0	0	0	0	0	0	0	0	0
JS061	1.66	0.0025	0	0.08	1.46	0	0.178	0.2	0.53	0.186
JS062	0	0	0	0	0	0	0	0	0	0
JS063	0.528	0	0	0.0238	0.029	0	0.0376	0.0114	0.162	0.0234
JS064	2.35	0	0	0.565	1.29	0	0.18	0.195	0.945	0.2
JS065	0.096	0	0	0.0184	0.57	0.003	0.015	0.007	0.1	0.006
JS066	0.0359	0.0077	0	0.0604	0.0971	0.0025	0.0146	0.0072	0.0163	0
JS067	0.87	0.085	0	0.145	2.74	0.0127	0.145	0.0313	0.765	0.025

Supplementary Table S2 Mean and Normalized Risk Values of Various Chlorinated Hydrocarbons

Pollutant	Carcinogenic Risk (non-boiled)		Non-Carcinogenic Hazard (non-boiled)		Carcinogenic Risk (boiled)		Non-Carcinogenic Hazard (boiled)	
	Mean	Standardized	Mean	Standardized	Mean	Standardized	Mean	Standardized
Trichloroethylene	3.79E-04	3.54E-04	2.46E+02	2.30E+02	1.57E-04	1.46E-04	1.07E+02	1.00E+02
Chloroform	1.99E-05	2.62E-04	8.37E-01	1.10E+01	9.25E-06	1.21E-04	3.41E-01	4.47E+00
Carbon Tetrachloride	6.36E-07	5.45E-04	3.18E-02	2.75E+01	2.71E-07	2.28E-04	1.31E-02	1.13E+01
Vinyl Chloride	7.92E-04	5.46E-03	5.40E+00	3.72E+01	3.20E-04	2.20E-03	2.25E+00	1.55E+01
1,1-Dichloroethane	9.13E-05	4.54E-05	1.14E+00	5.66E-01	3.94E-05	1.96E-05	4.84E-01	2.40E-01

1,2-Dichloroethane	3.97E-06	6.95E-04	1.07E-01	1.88E+01	1.62E-06	2.83E-04	4.54E-02	7.94E+00
1,1,2-Trichloroethylene	3.74E-05	4.36E-04	4.65E+00	5.41E+01	1.53E-05	1.78E-04	3.25E+00	3.79E+01
1,1-Dichloroethylene	——	——	1.05E-01	2.48E+00	——	——	4.99E-02	1.17E+00
1,2-Dichloroethylene	——	——	2.63E+01	3.65E+01	——	——	2.63E+01	1.48E+01
Tetrachloroethylene	8.87E-07	1.64E-05	1.02E+00	1.89E+01	3.75E-07	6.95E-06	4.35E-01	8.06E+00

Supplementary Table S3 Risk Control Values for the Parameters

Risk control value	TCE	$CHCl_3$	CCl_4	VC	1,1-DCA	1,2-DCA	1,1,2-TCA	1,1-DCE	1,2-DCE	PCE
Concentrations of pollutants at carcinogenic risk (mg/L)	0.00288	0.00428	0.00190	0.00018	0.02328	0.00146	0.00233	—	—	0.06318
Concentrations of contaminants at non-carcinogenic risks (mg/L)	0.00461	0.09214	0.03686	0.02764	1.84284	0.03686	0.03686	0.46071	0.02764	0.05529
Exposure period under carcinogenic risk (a)	0.067	1.383	41.126	0.032	0.289	8.296	0.677	—	—	29.297
Exposure period at non-carcinogenic risk (a)	0.108	29.773	799.680	4.765	22.879	161.315	10.726	270.617	0.958	25.635
